# Nonisothermal Thermogravimetric Analysis of Thai Lignite with High CaO Content

**DOI:** 10.1155/2013/216975

**Published:** 2013-10-23

**Authors:** Pakamon Pintana, Nakorn Tippayawong

**Affiliations:** Department of Mechanical Engineering, Faculty of Engineering, Chiang Mai University, Chiang Mai 50200, Thailand

## Abstract

Thermal behaviors and combustion kinetics of Thai lignite with different SO_3_-free CaO contents were investigated. Nonisothermal thermogravimetric method was carried out under oxygen environment at heating rates of 10, 30, and 50°C min^−1^ from ambient up to 1300°C. Flynn-Wall-Ozawa (FWO) and Kissinger-Akahira-Sunose (KAS) methods were adopted to estimate the apparent activation energy (*E*) for the thermal decomposition of these coals. Different thermal degradation behaviors were observed in lignites with low (14%) and high (42%) CaO content. Activation energy of the lignite combustion was found to vary with the conversion fraction. In comparison with the KAS method, higher *E* values were obtained by the FWO method for all conversions considered. High CaO lignite was observed to have higher activation energy than the low CaO coal.

## 1. Introduction

A significant proportion of the electricity generated in the world comes from pulverized coal-fired utility boilers. Thailand is the second largest coal-producing country in Southeast Asia. Over 80% of Thailand's total annual production comes from the Mae Moh surface mine in northern Thailand. Mae Moh coal is low grade lignite, containing relatively high percentages of ash and sulfur. The Mae Moh mine produces around 16 million tons of lignite per year to meet the 2400 MW capacity of Mae Moh's mine-mouth thermal power plant [1]. According to the Thailand power development plan for the period 2012–2030, electricity supply from coal remains a significant contributor to the future energy mix [2]. Considerable reserves of poor quality coal will have to be utilized efficiently with minimum impact to humans and the environment. 

Characteristics of the lignite in the different regions of the Mae Moh mine vary greatly from the design coal. Blending of the coals is normally adopted to increase fuel flexibility, improve the performance of coals, and extend the range of usable coals [3]. The low grade coals can be mixed with the better grade ones to meet the power plant input fuel requirement, thus avoiding deterioration in the thermal performance of the power plant. For the current practice at the power plant, the mixed coal must have sufficiently high heating value under limitation of sulfur content, and CaO in ash on a SO_3_-free basis must not exceed 23% w/w to prevent possible slagging problems [4, 5]. A recent survey of the lignite available in the remaining reserve suggests that coal blending may face mounting challenges because a major fraction of the coal mined will represent lignite with more than 40% CaO. Plant operations with lignite that contains increasingly higher CaO may be inevitable in the future.

A better understanding of the thermal decomposition and burning characteristics of this poor quality coal is crucial for safe and efficient operation of the boilers. Research on coal combustion may be performed using laboratory scale equipment. Thermogravimetric (TG) analysis is a simple and practical technique used to determine a material's thermal stability and its fraction of volatile components by monitoring the weight change that occurs as a specimen is heated. The rate of mass loss as a function of temperature and time is measured, and the kinetic parameters in the thermal decomposition reaction are estimated [6, 7]. TGA has been used to study the combustion and kinetic behavior of various coal samples. It is commonly performed by an isothermal or nonisothermal procedure to understand the degradation behavior of coal and to estimate the kinetic parameters of char combustion or gasification [8]. A number of studies have been conducted on the kinetics of thermal decomposition of coals [9–13]. As for low rank coals, several recent reports have been published on the thermal decomposition kinetics [13–21]. 

There appears to be very few published reports on the combustion kinetics of poor quality coal [13]. Thai lignite is considered to be of poor quality because of its high sulfur (up to 5.5%) and ash content and because there is a large amount of SO_3_-free CaO in its ash. So far, kinetic study on Thai lignite has been very rare [22, 23]. Furthermore, coals available currently and in future have different properties and compositions from those investigated in earlier reports. Nonisothermal isoconversional approach was not yet studied to any great extent. Therefore, in this paper, the burning characteristics of the lignite with different CaO contents were considered. Nonisothermal TG data at different heating rates was correlated with the kinetic parameters. Both Flynn-Wall-Ozawa (FWO) and Kissinger-Akahira-Sunose (KAS) methods were applied. The intrinsic kinetic from the TG analysis of the poor quality lignite will be useful for the design, modeling, planning, and understanding of the future operations of the power plant.

## 2. Material and Methods

### 2.1. Sample Preparation

The two different grades of lignite, based on the SO_3_-free CaO content, were studied for thermal analysis. They were collected from different regions of the Mae Moh mine reserve and typically characterized as low and high CaO lignite samples. Prior to size reduction, they were dried in ambient. The samples were subsequently crushed in a hammer mill and sieved to the size of about 60 mesh. The ground lignite samples were dried and sent for analysis. The coal samples were analyzed in accordance with ASTM standards for proximate analysis (ASTM D 3176-07a, D 3173-03 (2008), D 3174-04, and D 3175-07), ultimate analysis (ASTM D 3176-89), sulfur in coal (ASTM D 5016-08), heating value (ASTM D 5865-10a), and ash composition (ASTM D 4326-04). 

### 2.2. Thermal Measurements and Kinetics Modeling

Thermal analysis of lignite samples was carried out in a computer-controlled Perkin Elmer TGA 7 thermal analyzer. Prior to the experimental runs, the instrument was calibrated for precise temperature and weight readings. A quantity of 5.2 ± 0.1 mg of coal sample was used for each test. The digital microbalance is sensitive to 0.1 *μ*g. Nonisothermal experimental runs were performed at three different heating rates of 10, 30, and 50°C min^−1^ under oxygen atmosphere. The flow rate of the carrier gas was maintained at 50 cm^3^ min^−1^. The furnace temperature operated from ambient up to 1300°C. These dynamic runs were carried out on a platinum pan. The continuous records of weight loss and the weight loss rate with temperature were obtained. Thermal degradation behaviors were shown as TG and differential TG (DTG) profiles. They were also used as data for kinetics modeling.

For kinetics modeling, nonisothermal isoconversional approach was adopted in studying the kinetics of the Thai lignite. The approach has been shown to be able to estimate kinetic parameters without modeling assumptions [24, 25]. The kinetics of the thermal decomposition based on the rate equation for solid state decomposition processes [14] can be written as follows:

(1) $$\begin{array}{c} \frac{dx}{dt}=kf\left(x\right), \end{array}$$

where *x* is the decomposed fraction of solid at time  *t*, *f*(*x*) is a function of *x* depending on the reaction mechanism, and *k* is the rate constant given by the Arrhenius equation for nonisothermal chemical reaction as

(2) $$\begin{array}{c} k=k_{0}\exp\left(\frac{-E}{RT}\right), \end{array}$$

where *A* is the preexponential factor, *E* is the apparent activation energy, *R* is the universal gas constant, and *T* is the absolute temperature. Replacing *k* with the Arrhenius equation gives

(3) $$\begin{array}{c} \frac{dx}{dt}=A\exp\left(\frac{-E}{RT}\right)f\left(x\right). \end{array}$$

The decomposed fraction, *x*, found convenient to express a reaction by using a certain function *f*(*x*), is defined in terms of the normalized change in mass of the sample as

(4) $$\begin{array}{c} x=\frac{w_{0}-w_{t}}{w_{0}-w_{f}}, \end{array}$$

where *w*_0_ is the initial weight, *w*_*f*_ is the final weight, and *w*_*t*_ is the weight at time *t* of the sample analyzed by the nonisothermal TG analysis.

The FWO method is [26]

(5) $$\begin{array}{c} \ln\beta =\ln\left[\frac{AE_{x}}{Rg\left(x\right)}\right]-5.331-\frac{E_{x}}{RT_{x}}, \end{array}$$

where *β* is the heating rate and *g*(*x*) is the integral form of the *f*(*x*). At a constant conversion, the plot of ln⁡*β* versus 1/*T* obtained at several heating rates is approximated to be a straight line whose slope allows evaluation of the activation energy. The intercept can be obtained from the straight line and assumed to be first order of reaction from *f*(*x*) = (1 − *x*), and *g*(*x*) = −ln⁡(1 − *x*), and second order of reaction from *f*(*x*) = (1−*x*)^2^ and *g*(*x*) = (1−*x*)^−1^ − 1, and third order of reaction from *f*(*x*) = (1−*x*)^3^ and *g*(*x*) = 0.5⌊(1−*x*)^−2^ − 1⌋ [27–29].

The KAS method is as follows [26]:

(6) $$\begin{array}{c} \ln\left(\frac{\beta }{T_{x}^{2}}\right)=\ln\left[\frac{AR}{E_{x}g\left(x\right)}\right]-\frac{E_{x}}{RT_{x}}. \end{array}$$

The activation energy can be calculated from plotting ln⁡⁡(*β*/*T*_*x*_^2^) against 1/*T* and from the preexponential factor from the intercept of the resulting straight line.

## 3. Results and Discussion

### 3.1. Coal Characteristics

A comparison of the results from the proximate and ultimate analyses and the calorific values of the two lignite samples, as well as their ash composition are shown in Table 1. It can be clearly seen that Mae Moh low rank coal is of poor quality, with extremely high moisture content (35–40% on as-received basis) and high S content (3.3–5.5% on dry basis). Their ash content is in the range of 17–20% on dry basis. While both low and high CaO lignite samples show similar calorific values, volatility, and CHO contents, their S contents differ markedly. As far as the amount of SO_3_-free CaO in ash is concerned, the high CaO lignite has about three times more than the low CaO sample. The base-to-acid ratio of the former is about twice that of the latter, indicating higher slagging tendency.

### 3.2. Thermal Behaviors

**Figure 1:**
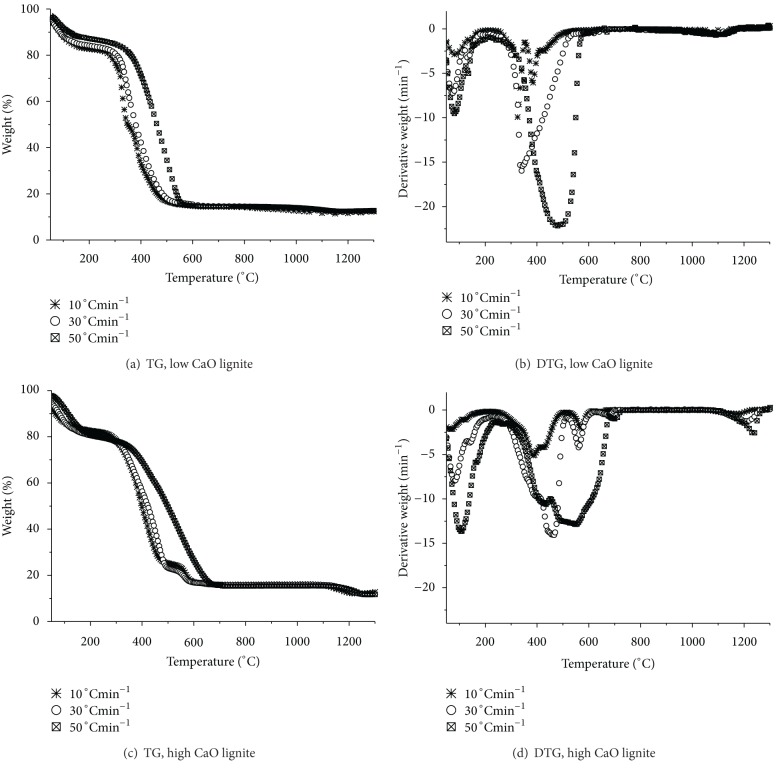
Thermal degradation profiles of low and high CaO samples at different heating rates.

The thermal characteristics of the coals were shown as change in weight with temperature (TG) and rate of weight loss (DTG) profiles.  Figure 1 shows these degradation profiles of low and high CaO lignite samples at different heating rates. Continuous weight loss was evident. Both the lignite samples show similar TG patterns, suggesting that the same kinds of reactions occurred for all the heating rates considered, but the temperature ranges were different such that the TG curves shifted to higher temperatures as the heating rate increased. Three major weight loss stages can be characterized from the TG curves, corresponding to (i) the release of moisture in the sample, (ii) the release of volatile matter and combustion of char, and (iii) the decomposition of the mineral matter in the sample [16–18, 30]. For a given heating rate, the low CaO samples appeared to exhibit sharper changes in the TG slope than the high CaO coal, consistent with the higher negative peaks of the DTG profiles. These observations were true for the first two stages. Higher heating rate was found to show wider temperature range for mass loss. The first stage of the low CaO lignite was from 50 to about 200°C, while the first stage of the high CaO lignite covered from 50 to about 250°C. The main release of organic components and the char combustion stage for the low and high CaO lignite samples were from 200 to 575°C and 300 to 700°C, respectively. The onset of the devolatilization was delayed for the higher CaO content lignite. Thermal degradation at this second stage of mass loss showed the peak rate for the low CaO lignite sample to be higher than that for the high CaO coal. From a comparison of the thermal degradation patterns, the reactions were found to take place at higher temperatures as the CaO content in the lignite increased. Since both the coal samples have similar amounts of volatile matter, they would be expected to exhibit similar thermal degradation at similar ranges of temperature [18]. It was not yet clear if the presence of the higher CaO content may have contributed to this slight difference. Nonetheless, it should also be noted that, at a given stage of weight loss, the high CaO coal showed lower mass loss rate than the low CaO coal. Hence, the former was less reactive than the latter, if we take into account the direct proportionality between the peak loss rate and the reactivity. The findings implied that higher CaO containing lignite may require higher temperatures to react, but at slower rates.

### 3.3. Kinetic Analysis

**Figure 2:**
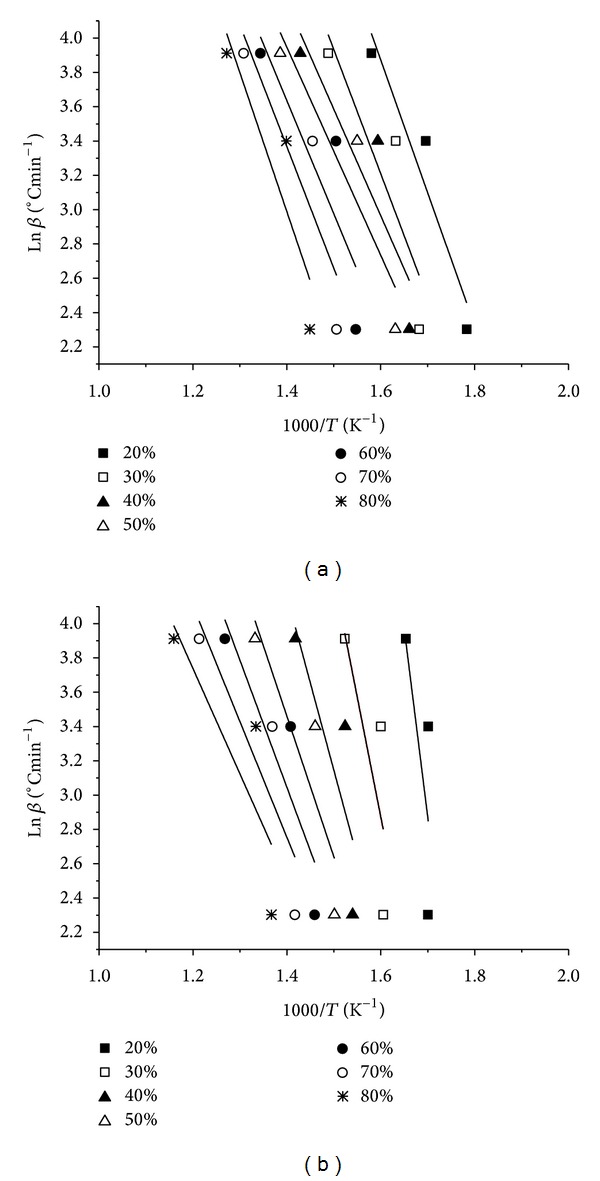
Isoconversional determination of the kinetic parameters based on the FWO method for (a) low CaO and (b) high CaO lignite samples.

**Figure 3:**
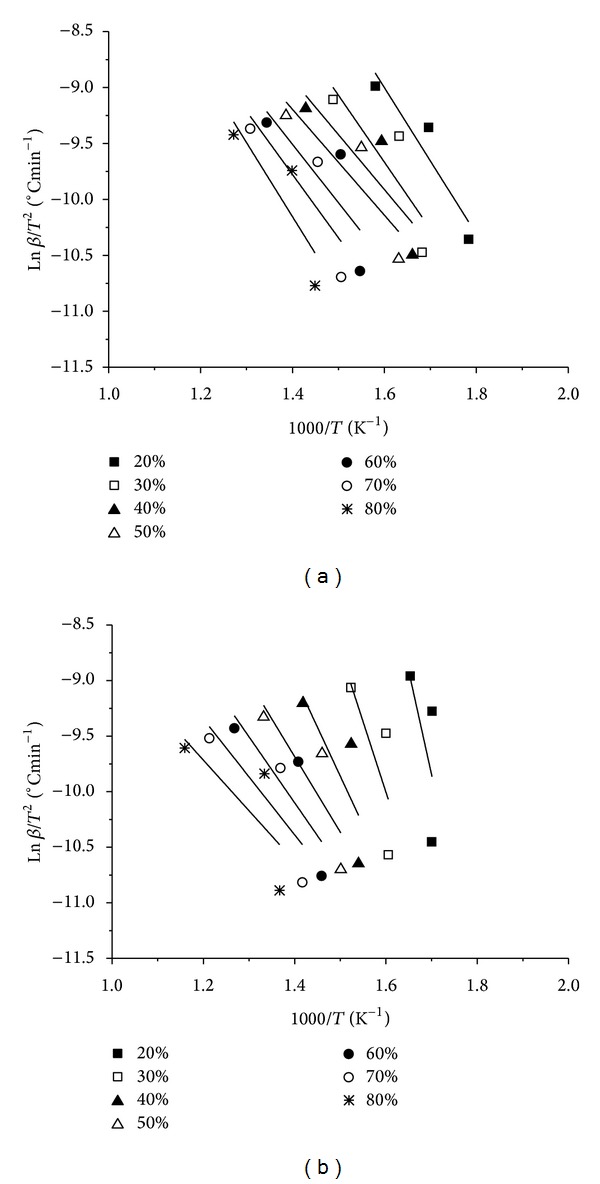
Isoconversional determination of the kinetic parameters based on the KAS method for (a) low CaO and (b) high CaO lignite samples.

**Figure 4:**
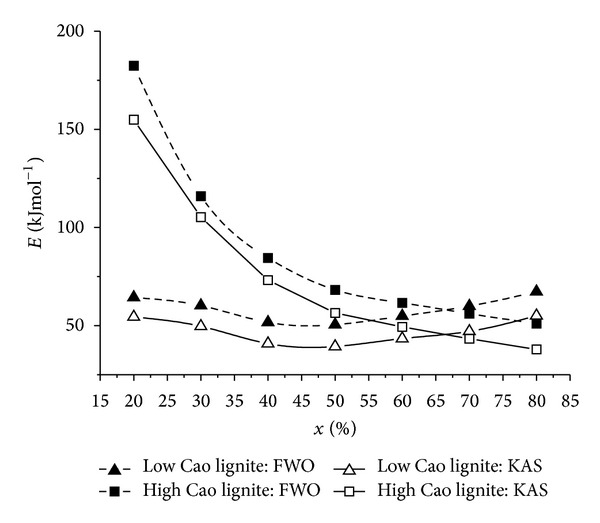
Dependency of apparent activation energy (*E*) on conversion (*x*), determined using the FWO and KAS methods for low and high CaO lignite samples.

The plot of ln⁡⁡*β* versus 1/*T* by the FWO method is shown in Figure 2. Three different heating rates were used, generating different Arrhenius plots at various conversions, from 20 to 80%. The apparent activation energy and preexponential factor were estimated from linear regression analysis for each conversion. Similarly, Figure 3 shows the relationships between ln⁡⁡(*β*/*T*_*x*_^2^) and 1/*T* by the KAS method at various conversions, from 20 to 80%. Straight lines were approximated and used to calculate relevant kinetic parameters. It should be noted that the quality of fittings was not very good. This may be caused by model and experimental system inadequacy and heat transfer limitations. Nonetheless, the variation of the activation energy with the extent of conversion is depicted in Figure 4. Similar relationships between the activation energy and conversion were observed between those obtained by the FWO and the KAS methods for the respective lignite sample. Within the conversion rates considered, the *E* values obtained from the FWO method appeared to be slightly higher than those from the KAS method. This was due to the distinctive linear approximation to the temperature integral. This observation was in agreement with those reported by Sis [18] and Xiao et al. [26]. Regarding the two lignite samples, the *E* values based on the FWO method for the high CaO coal were found to vary radically over the range of the conversion rate, decreasing from around 180 KJ mol^−1^ at *x* = 0.2 to 50 kJ mol^−1^ at *x* = 0.8, while those for the low CaO coal appeared to fluctuate slightly within the narrow band, about 65 ± 10 kJ mol^−1^. Since the extent of conversion was directly related to temperature, the observed change in the *E* values should be attributed to the different reaction rates of dehydration, devolatilization, char combustion, and decomposition of mineral matter [16, 17]. Conversions in the 0–20% and 80–100% ranges occur in the lowest and highest ranges, hence they are associated mainly with the moisture release and mineral matter decomposition, respectively. In this work, the kinetic parameters were obtained in 0.2–0.8 conversion range; therefore, they were related mostly to the volatile release and char combustion. The average values of *E* and *A* assuming the first and second order of reaction are summarized in Table 2. The parameter *A* obtained were markedly different for the FWO and KAS methods, even for the 1st order reaction. However, the average activation energies obtained were in similar magnitude. The values of *E* by the FWO and KAS methods were calculated to be 58.4, 47.3 and 88.5, 74.4, for the low and high CaO lignite samples, respectively. The discrepancy between the two lignite samples may be attributed to the diversity in the composition nature and the structure of the samples. The implication from the findings was that the high CaO lignite sample may be less homogeneous than the low CaO coal.

## 4. Conclusion 

In this study, the nonisothermal TG analysis of Thai lignite under highly oxidative environment was investigated at three different heating rates, up to 1300°C. The proximate and ultimate composition, sulfur in coal, calorific value, and ash composition of the low and high CaO lignite samples were analyzed. TG and differential TG curves were used to describe the different thermal degradation profiles for both the coal samples. Isoconversional kinetic analysis based on the FWO and KAS methods was found to be useful in evaluating the combustion kinetic parameters. It was observed that the apparent activation energy of coal combustion varied with the conversion fraction. The high CaO lignite was found to show higher activation energy than the low CaO coal.

## Figures and Tables

**Table 1:** Analysis results of Thai lignite samples.

Characterization	Low CaO lignite	High CaO lignite
Proximate analysis (% w/w, as-received basis)		
Moisture content	35.1	39.6
Volatile matter	28.2	27.6
Fixed carbon	25.8	20.9
Ash	10.9	11.8
Ultimate analysis (% w/w, dry basis)		
C	58.5	58.4
H	3.0	2.4
N	1.9	1.8
O	12.9	12.6
S	5.5	3.3
Heating value (MJ/kg, dry basis)		
HHV	23.7	22.6
LHV	23.1	22.0
Ash compositions		
Na_2_O	1.9	0.7
MgO	4.2	3.4
Al_2_O_3_	13.4	1.4
SiO_2_	21.1	16.6
P_2_O_5_	0.1	0.2
SO_3_	17.4	33.4
K_2_O	1.3	0.2
TiO_2_	0.3	0.1
Fe_2_O_3_	28.9	15.9
MnO_2_	0.1	0.1
CaO	11.4	28.2
CaO (SO_3_-free basis)	13.8	42.3
Base-to-acid ratio	1.37	2.68

**Table 2:** Calculated combustion kinetic parameters.

Method	Material		*n*	Average	s.d.
FWO	Low CaO lignite	*E* (kJ/mol)		58.40	6.35
		*A*	1st	1.30 × 10^−4^	1.04 × 10^−4^
			2nd	2.13 × 10^−4^	1.64 × 10^−4^
			3rd	3.98 × 10^−4^	3.08 × 10^−4^
	High CaO lignite	*E* (kJ/mol)		88.55	46.87
		*A*	1st	1.58 × 10^−5^	1.37 × 10^−5^
			2nd	2.03 × 10^−5^	1.72 × 10^−5^
			3rd	4.72 × 10^−5^	3.86 × 10^−5^

KAS	Low CaO lignite	*E* (kJ/mol)		47.25	6.30
		*A*	1st	1.70 × 10^−2^	1.03 × 10^−2^
			2nd	2.84 × 10^−2^	1.78 × 10^−2^
			3rd	5.51 × 10^−2^	4.18 × 10^−2^
	High CaO lignite	*E* (kJ/mol)		74.35	42.21
		*A*	1st	1.88 × 10^−2^	2.85 × 10^−2^
			2nd	4.14 × 10^−2^	7.04 × 10^−2^
			3rd	1.10 × 10^−1^	2.11 × 10^−1^
